# The Effect of Olive Leaf Extract on Upper Respiratory Illness in High School Athletes: A Randomised Control Trial

**DOI:** 10.3390/nu11020358

**Published:** 2019-02-09

**Authors:** Vaughan Somerville, Rachel Moore, Andrea Braakhuis

**Affiliations:** Discipline of Nutrition and Dietetics, Faculty of Medical and Health Science, The University of Auckland, Auckland 1023, New Zealand; rachel.moore1710@gmail.com (R.M.); a.braakhuis@auckland.ac.nz (A.B.)

**Keywords:** polyphenols, sport, immunity, athlete, respiratory, oleuropein, hydroxytyrosol

## Abstract

Upper respiratory illness (URI) has a major impact on both training and competition in an athletic setting. High school athletes are a sub-category who have reported higher illness rates than professional and sub-elite high school athletes of the same sport. Olive leaf extract (OLE) is an over-the-counter supplement that contains polyphenols, notably oleuropein and hydroxytyrosol, that have antiviral, antibacterial, anti-inflammatory and antioxidant properties that may reduce URI rates. Thirty-two high school students who play sport for the elite team at their school were recruited to a randomised controlled trial and allocated to a daily placebo or OLE (extent equivalent to 20 g of olive leaf, containing 100 mg oleuropein) supplementation for nine weeks during their competitive season. Twice weekly measures of wellbeing, training load and respiratory illness (sporting upper respiratory illness (SUPPRESS) questionnaire) were recorded at trainings, meetings or games. There was no significant difference in illness incidence (odds ratio (OR): 1.02 (95% confidence interval (CI) 0.21–4.44)), but there was a significant 28% reduction in sick days (OR: 0.72 (95% CI 0.56–0.93) *p*-value = 0.02) when supplemented with OLE. The dietary intakes of the athletes were sub-optimal with regard to immune support. OLE supplementation over a season did not significantly reduce URI incidence, but did decrease duration in high school athletes, potentially aiding return to play.

## 1. Introduction

When an individual exercises, there is a 3 to 72 hour subsequent period known as the ‘open window’ where they are susceptible to illness due to altered immune function and activity [1]. Consequently, upper respiratory illness (URI) is a prevalent health complaint for athletes [2,3]. URI episodes have a significant impact on training and performance with 19% and 31% of athletes reporting a decrease in or complete cessation of training, respectively [4]. School athletes are a specific cohort who, in addition to high training loads, have external contributors (e.g., academic and social demands) that are associated with increased stress and URI risk [5]. This is illustrated by the increased illness rates in the 2012 Winter Youth Olympic Games (8.4%) [2,6]. In addition, it has been reported that the respiratory illness rates in elite high school athletes is higher than other levels in the same sport [7]. 

With no effective front-line cure, treatment plans for URIs are centred on symptom alleviation [8]. Sports teams are therefore focusing on novel nutritional supplements that may decrease URI incidence [9,10,11,12]. A recent meta-analysis reported that flavonoids, a sub group of polyphenols, decreased URI incidence by 33% after at least 7 days’ supplementation [9]. Olive leaf extract (OLE) is an over-the-counter supplement containing a range of polyphenols, predominately oleuropein and hydroxytyrosol (HT). Oleuropein and HT have a range of mechanisms that may reduce URI incidence and duration. Firstly, oleuropein acts as an antioxidant with dose-dependent inhibition of the copper sulphate-induced oxidation of low-density lipoproteins (LDLs) [13]. Furthermore, oleuropein increases nitric oxide production in macrophages, increasing functional activity [14]. Oleuropein also has an effect on the aetiology of URIs by producing strong antimicrobial activity against gram-positive and gram-negative bacteria and antiviral activity against respiratory syncytial virus (RSV), a common URI virus [15,16,17]. 

Scientific and anecdotal evidence of the health benefits of OLE supplementation have led to significant attention and use [18]. To date, there is no research investigating the effect of OLE on URI incidence or duration. The objective of this study is therefore to determine the effect of OLE supplementation on URI incidence and duration in high school athletes.

## 2. Materials and Methods

### 2.1. Design

The study was a two-month parallel double-blind randomised controlled trial (RCT) conducted from May to July 2018. Participants were randomly allocated to OLE (extent equivalent to 20 g of olive leaf, containing 100 mg oleuropein) or placebo tablets (gluten-free corn starch). Both capsules were the same size and colour. Participants were required to take one tablet daily for two months and complete a questionnaire twice weekly. The participants were instructed to ingest the tablets at the same time every day. Participants were recruited by R.M. and subsequently randomly allocated by A.B. using a research randomiser (https://www.randomizer.org/#randomize). A.B. had no contact with participants and was not involved in data collection. The tablets were put into a white envelope with the participant number written by A.B. and distributed by V.S. and R.M. to ensure double-blinding. At completion, participants gave back the remaining tablets to measure compliance and asked which product they believed they were consuming. The primary outcome was to determine the effect of OLE on URI incidence and duration. Secondary outcomes were to determine the effect of OLE on different characteristics of wellbeing when training load was controlled for and the potential effect of nutritional status on URI incidence and duration. The trial and study design was registered with the Australian New Zealand Clinical Trials Registry (ACTRN12618000328279).

### 2.2. Participants

Participants were recruited from one West Auckland school in New Zealand in May 2018. To be included, participants had to be aged 16 to 19 years, attend school, play an elite sport for that school, be healthy and have not consumed polyphenol supplements (quercetin, propolis, turmeric, curcumin, etc.) in the three previous months. Participants were excluded if they had asthma or were smokers. All participants were advised to continue their usual training and diet regime (including other medicines and supplements). In the parallel-group randomised trial, a sample size estimation of 28 subjects was estimated based on an expected rate of 2.0 ± 1.0 URI episodes (mean ± SD) during training months [19], a target 33% reduction in the number of episodes (incidence reduction of 0.6 episodes), requiring a statistical power of 80%, and a Type I error of 5%. The reduction target is based on previous research indicating that dietary polyphenols decrease URI incidence by 33% (95% confidence interval (CI): 31%, 36%) following at least 7 days’ supplementation [9]. As a result, 32 participants were recruited to allow for a 10% drop-out rate. The study was conducted in accordance with the Declaration of Helsinki and ethical approval was obtained from the Health and Disabilities Ethics Committees (Reference number 18/NTA/38). All subjects gave their informed consent for inclusion before they participated in the study.

### 2.3. Questionnaires

Participants were required to complete a questionnaire twice a week before their respective trainings. The questionnaire was broken down into (1) wellbeing, (2) training load and (3) respiratory illness (validated sporting upper respiratory illness (SUPPRESS) questionnaire). Participants also completed three random 24-h food recalls to assess dietary and polyphenol intake.

2.3.1 Wellbeing

Wellbeing was investigated in five categories: Soreness, stress, sleep quality, fatigue and satiety. Participants were asked to rate a question relating to each category on a scale of 1–7. The questions were designed after piloting and consultation from strength and conditioning coaches (*n* = 4), key players (*n* = 2) and school-team coaches (*n* = 1). The questions included:

1. How sore are you today?
2. How stressed are you currently?
3. Rate last night’s quality of sleep.
4. How physically fatigued are you today?
5. How hungry have you been recently?

#### 2.3.2. Training Load

Each questionnaire required participants to report game time, cardio training and resistance training undertaken since the last questionnaire. No information about training intensity or heart rate was collected. 

#### 2.3.3. Upper Respiratory Illness

This section comprised the validated SUPPRESS questionnaire [20]. Participants were asked if they had a cold and then to rate four respiratory symptoms (nasal, sore throat, cough and sneezing) that they may have experienced over the last 48 h on a 0–3 scale of increasing severity and impact. A URI was codified as per the SUPPRESS protocol. 

#### 2.3.4. 24-Hour Food Recalls

Participants completed three randomly allocated 24-h food recalls. Each recall was examined by one nutritional researcher (V.S. or R.M.) who asked participants further questions to elucidate details (i.e., amount, brand, cooking process) where insufficient information was provided. At the completion of the study all participants were sent an automated self-administered 24-h (ASA24) questionnaire. 

### 2.4 Statistical Analysis

Participant characteristics were collected and presented as the mean ± standard deviation (SD) where appropriate. Data for incidence and duration were reported as the odds ratio (OR) with a 95% CI. Data were analysed via GraphPad Prism (version 7.03, GraphPad Software, La Jolla, California, USA, www.graphpad.com) using a two-sided Fisher’s exact test with significance reported if *p* < 0.05.

#### 2.4.1. URI Incidence

The number of URI episodes per person was calculated by the SUPPRESS protocol. The total number of people who experienced a URI was also calculated. If a participant failed to fill in a questionnaire on a certain date and the previous questionnaire had classified them as having a URI episode, the missed questionnaire was also counted as the same URI episode. Conversely if the previous questionnaire had not codified a URI episode, the missed questionnaire was similarly coded. 

#### 2.4.2. URI Duration

To calculate the duration of URI episodes, where the participant symptom score was ≥4 on day 1 of the URI episode, 2.5 days were added to the duration to reflect the symptoms lasting more than 48 h as per the questionnaire. When the symptom score was <4, 1 day was added to reflect the period in which a participant could self-identify as having a cold. Missing data was similarly recorded as the above illness.

#### 2.4.3. Secondary Outcomes

The effect of the intervention on aspects of wellbeing between the two groups was analysed using the sportscience pre-post parallel-groups controlled trial spreadsheet, using day 0 as the baseline (http://sportscience.sportsci.org/). Training load was added as a cofounding variable and was taken as a sum of game time, cardio training and resistance training. Data were entered into the spreadsheet and presented as the percentage difference in the means and an associated odds ratio clinical inference statement. The outcomes have been interpreted using the odds ratio clinical inference statement. This approach takes into account the risks of benefit and harm, rather than the line of no effect, to declare a clear or unclear outcome. The wellbeing scores were deemed clear if the odds ratio was >66 between the OLE and control groups. 

#### 2.4.4. 24-Hour Food Recalls

The 24-h food recalls were entered into FoodWorks Professional (Xyris Ltd, Version 2015, Xyris Software Australia Pty Ltd, Brisbane, Australia). For the ASA24, data were collected and analysed using the ASA24 Dietary Assessment Tool, (Version 2016, National Cancer Institute, Bethesda, MD, USA) (https://epi.grants.cancer.gov/asa24). From this the daily average total energy intake (kJ·day^−1^), the macronutrient and the micronutrient intake were extracted. Over- and under-reporters were identified using Tukey’s fences method of interquartile range (IQR) to create a lower and upper cut-off ((quartile 1 − (1.5 × IQR)) and (quartile 3 + (1.5 × IQR)), respectively) [21]. The polyphenol intake was analysed using the PhenolExplorer Food Composition Database (http://phenol-explorer.eu/) and was recorded in the database if a food serving contained >30 mg of total polyphenols. Foods high in polyphenol content were identified by each reviewer and inserted into the polyphenol database. Given the subjective nature of polyphenol food selection, one 24-h recall was examined for each participant by two researchers independently (V.S. and A.B.) for polyphenol intake. Discussion was conducted when researchers disagreed.

## 3. Results

### 3.1. Population Characteristics

**Figure 1:**
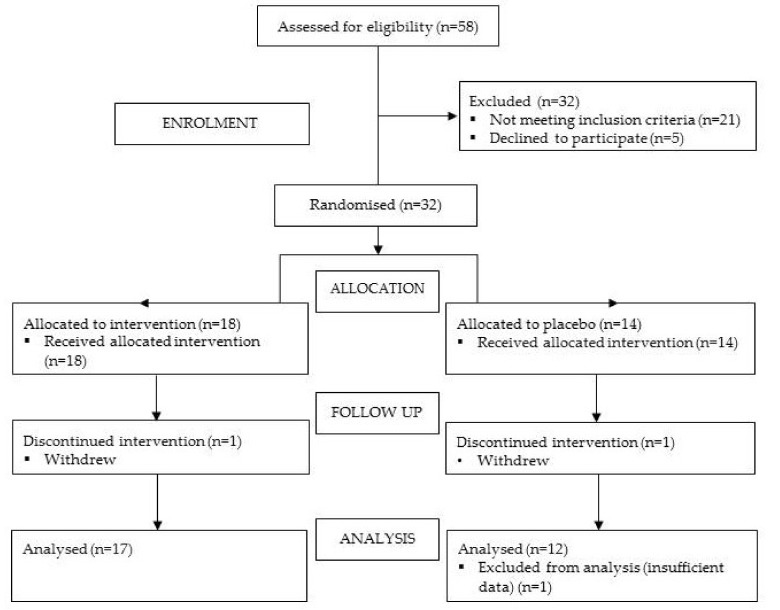
CONSORT flowchart.

Thirty-two students were recruited from three different sport codes (hockey, football and netball). Twenty-two of these participants were female. The characteristics of each group are shown in Table 1. Throughout the study three participants (OLE *n* = 1; placebo *n* = 2) did not complete the questionnaire a sufficient number of times (25%) or withdrew (*n* = 2) and were excluded from the analysis (Figure 1). Sixty percent of each group correctly identified whether they were on OLE or a placebo, therefore the blinding appeared effective. Of note is the difference in the mean training load between the groups. One person allocated to the OLE group was also a competitive swimmer, training substantially more, which may have accounted for the discrepancy.

### 3.2. URI Incidence and Duration

There were 17 participants in the study who experienced a total of 26 URI episodes. There was no significant difference in incidence; however, there was a significant 28% reduction in sick days (Table 2). The average duration of each URI episode was 9.7 and 12.3 days in the OLE and control groups, respectively. Four participants in the OLE group experienced more than one URI episode during the intervention, compared with two in the control group. Of these (*n* = 6), three episodes was the maximum number experienced. There was no significant effect of OLE incidence in either males or females when separated; however, there was a significant reduction in sick days for females (OR: 0.57 (95% CI 0.43–0.77), *p* value < 0.01) and an increase for males (OR: 1.96 (95% CI 1.01–3.85), *p* value < 0.05). 

### 3.3. Secondary Outcomes

OLE had a possibly harmful and a likely harmful effect on soreness and stress, respectively, when training load was controlled. The remaining categories were unclear and needed more data.

Dietary intake is summarised in Table 3, with no significant differences between the groups. One participant was removed from the control group based on the Tukey fence method due to over-reporting. One recall from a participant in the OLE group was removed as they had a gastro-intestinal illness the day before. 

### 3.4. Adverse Effects

Five participants reported adverse effects, of which four were on OLE. Three participants on OLE reported stomach aches and headaches, and the other reported bad skin and acne. The participant on the placebo reported vomiting, stomach aches, rash and period cramps. 

## 4. Discussion

To the authors’ knowledge, this is the first study investigating the effects of OLE supplementation on any athlete cohort relating to health and illness indices. The primary outcome of the study suggests that OLE has little effect on URI incidence but reduces sick days by 28% in high school athletes. Secondly, both cohorts had sub-optimal carbohydrate intake, which may have mitigated any further effects of OLE supplementation [22,23,24]. This study highlights that URIs are a problem in high school athletes, with at least 50% experiencing one or more URIs over a nine-week period, and 10% experiencing three. Although OLE did not decrease URI incidence, further research should investigate other individual and polyphenol combinations that may decrease incidence. 

Although both groups had nearly equal gender distribution, overall the study had more women (66%) than men, which may have influenced the outcomes. One previous study on athletes reported that the URI incidence in females was insignificantly higher than in males (0.6 ± 0.8 in males and 0.8 ± 1.0 in females); however, the duration was longer for females (6.8 ± 7.1 days for females and 4.7 ± 5.0 days for males (*p* = 0.016)) [25]. Conversely, in the 2012 Winter Youth Olympics women had an increased overall illness rate (risk ratio = 1.84 (95% CI 1.21 to 2.78)); however, this was not separated into URIs [6]. In this study, there was no significant effect of OLE on incidence when gender was controlled for (*p* > 0.05), but interestingly there was a significant reduction in illness days in the females supplemented with OLE, but an increase in males (OR: 0.57 (95% CI 0.43–0.77) and 1.96 (95% CI 1.01–3.85), respectively). Stress and menstrual cycle are known influencers of immune parameters, including natural killer cells and CD4^+^:CD8^+^ ratio, and it is possible OLE has an improving effect on URI risk in women, ameliorating menstrual immune dysfunction [26]. Additional research should be conducted in single gender populations to elucidate this potential effect.

Interestingly, the majority of studies conducted investigating whether other nutritional supplements (vitamin D, vitamin C probiotics, bovine colostrum protein, flavonoids and β-glucan) decrease URI in athletes have shown no significant decrease in duration irrespective of a decrease in incidence [9,10,27,28,29,30], although vitamin C appears to decrease sick days while not influencing the incidence [31]. This difference could be explained by the effects of OLE, which include an increase in the functional activity of macrophages and in antimicrobial and antiviral activity [14,15,16,17]. Rather than alleviating the altered immune function that occurs post-exercise, OLE may have an effect once a pathogen has entered the host, preventing further replication and improving the host’s defence, thus reducing duration. 

Four participants on OLE supplementation experienced side effects. No serious adverse effects have been reported in OLE studies; however, other side effects have been reported, notably allergic reactions in those who have an allergy against the plant species (Oleaceae) [32]. Studies in mice have reported no adverse effects with acute oleuropein doses at 1000 mg·kgBW^−1^, including no toxicity of its breakdown products (HT and elenolic acid) at this dosage [33,34]. Despite recent studies on OLE supplementation for humans, which reported no increased adverse effects, more research is needed examining the safety in humans and potential long-term effects [35,36,37,38]. The authors recommend that the product is tolerable to the majority; however, as sport requires optimum performance, if any side effects are perceived by the athlete, supplementation should cease immediately. 

Nutritional intake can also be an important confounding variable when it comes to athletes and illness rates, especially in sports that require elements of leanness. Recent statements by the International Olympic Committee (IOC) and American College of Sports Medicine (ACSM) have remarked that athletes with low energy availability (LEA) have an increased risk of infections and illnesses, especially noting women and the female triad [22,23]. Both cohorts consumed less than the 5 to 7 g·kg^−1^·day^−1^ of carbohydrates recommended by the IOC for athletes exercising at this level [24]. The authors propose that the athletes may be in an immune-compromised state due to a lack of carbohydrates and subsequent LEA, which OLE supplementation is unlikely to offset. In addition to potential OLE supplementation, there is a need to educate sports people at this level regarding adequate macro and micronutrient intake to ensure optimal performance and health.

There are limitations in this study—notably, training intensity was not measured. Training intensity is an important variable in the extent and duration of the ‘open window’ [1]. This study included team sports, therefore an individual’s game time may have been the same but they had different demands and intensity profiles based on their position. If accounted for, this may have elucidated an effect of OLE not otherwise detected. 

It is also noted that compliance is not perfect in either group. As compliance analysis was undertaken at completion, the authors were not able to track what days or periods that participants missed. OLE has a rapid bioavailability with conjugated HT metabolites peaking approximately 1 hour after consumption [39]. Once ingested, oleuropein undergoes extensive hydrolysis during Phase I metabolism, releasing more HT, and then undergoes sulfation and glucuronidation during Phase II [40]. Consequently, the majority of oleuropein consumed circulates as other conjugated metabolites [40]. Research using another OLE product reported that consuming 76.6 or 51.1 mg of oleuropein (in OLE capsule form) resulted in peak plasma concentrations of 0.60 ± 0.37 and 0.52 ± 0.24 ng·mL^−1^, respectively [39]. It is noted that the liquid version of OLE has a significantly higher peak of metabolites; however, the liquid has a very strong and bitter taste which is likely to decrease compliance even further [39]. Both oleuropein and HT metabolites are excreted in urine within 24 h, so daily supplementation is needed to ensure circulating levels [41]. If a participant missed a few days consecutively it may have attenuated any effect of OLE supplementation. 

## 5. Conclusions

This is the first study investigating the effect of OLE on URI incidence and duration in school athletes. OLE had no significant effect on incidence but significantly reduced the number of sick days by 28%. A reduction in the number of days would likely aid in ‘return to play’ and potentially improve performance. High school athletes should be educated on adequate intake of nutrients to support immune function, mainly concerning carbohydrates, prior to any consideration of supplementation. This research shows that OLE supplementation could be used to alleviate the effect of URI on high school athletes; however, adequate food-based nutrition and potentially other polyphenol products should still be investigated to decrease incidence.

## Figures and Tables

**Table 1:** Population characteristics of included participants.

	Olive Leaf Extract (OLE) (*n* = 17)	Control (*n* = 12)
Age (±SD)	16.5 years (±0.5)	16.5 years (±0.5)
Weight (±SD)	69.2 kg (±13.0)	64.3 kg (±7.9)
Height (±SD)	1.73 m (±0.07)	1.73 m (±0.10)
Male	35%	33%
Wellbeing (±SD)	65.6% (±12.7)	64.8% (±12.5)
Game Time (±SD)	137 min·week^−1^ (±117)	112 min·week^−1^ (±114)
Cardio (±SD)	182 min·week^−1^ (±372)	99 min·week^−1^ (±72)
Weight (±SD)	19 min·week^−1^ (±35)	37 min·week^−1^ (±72)
Compliance (%)	74.7	83.8

**Table 2:** Effect of olive leaf extract (OLE) on upper respiratory illness (URI) incidence and sick days.

	OLE (*n* = 17)		Control (*n* = 12)	
	N	Proportion	N	Proportion
Incidence	10	0.59	7	0.58
Sick Days	145.5	0.16	135.5	0.20
	N		N	
Episodes	15		11	

Incidence odds ratio = 1.02 (95% CI 0.21–4.44) (*p* value > 0.05); sick day odds ratio = 0.72 (95% CI 0.56–0.93) (*p* value = 0.02).

**Table 3:** Population characteristics of included participants.

		OLE (*n* = 16)	Control (*n* = 11)
	Total Energy (±SD)	8209.9 (±3351.3) kJ·day^−1^	7826.9 (±2684.9) kJ·day^−1^
Macronutrient			
	Carbohydrate (±SD)	3.67 (±2.00) g·kgBW^−1^·day^−1^	3.58 (±1.41) g·kgBW^−1^·day^−1^
	Protein (±SD)	1.28 (±0.61) g·kgBW^−1^·day^−1^	1.34 (±0.39) g·kgBW^−1^·day^−1^
	Fat (±SD)	0.91 (±0.46) g·kgBW^−1^·day^−1^	1.04 (±0.47) g·kgBW^−1^·day^−1^
Micronutrient			
	Vitamin C (±SD)	94.50 (±69.02) mg·day^−1^	82.40 (±69.85) mg·day^−1^
	Vitamin D (±SD)	1.66 (±1.21) µg·day^−1^	2.06 (±1.50) µg·day^−1^
	Selenium (±SD)	54.40 (±19.96) µg·day^−1^	59.43 (±21.51) µg·day^−1^
	Iron (±SD)	15.17 (±9.60) mg·day^−1^	11.41 (±3.58) mg·day^−1^
	Zinc (±SD)	11.54 (±5.74) mg·day^−1^	10.21 (±3.82) mg·day^−1^
	Polyphenol (±SD)	115.60 (±176.76) mg·day^−1^	69.75 (±55.63) mg·day^−1^

kgBW = kg of body weight.
